# ‘All-in-one’ strategy for metalla[3]catenanes and ring-in-ring complex

**DOI:** 10.1093/nsr/nwaa235

**Published:** 2020-09-23

**Authors:** Xian-He Bu

Research into mechanically interlocked molecules, such as catenanes, rotaxanes and molecular knots, has been gaining momentum in recent decades, not only because of their intriguing structures and topological importance, but also because of their important applications as molecular machines and nanoscale devices [1,2]. In addition to traditional organic macrocycles based on covalent bonds, organometallic macrocycles or rectangles based on coordination self-assembly are promising alternative building blocks for the construction of molecular interlocked structures [3]. Some complicated interlocked molecules based on a reversible coordination bond have been realized by one-step processes. Obviously, the realization of this comprehensive protocol requires careful selection and elaborate design of the precursor and ligand. Despite extensive research and several stunning breakthroughs in the synthesis of interlocked molecular species, linear [3] catenanes and the ring-in-ring complex are exceedingly rare, and their targeted synthesis remains a formidable challenge [4].

Recently, Prof. Guo-Xin Jin from Fudan University (Shanghai, China) made exciting progress and developed coordination self-assembly of metalla[3]catenanes, molecular Borromean rings (BRs) and a ring-in-ring complex using a simple π-donor unit [5].

In this work, the authors used bithiophenyl groups as building blocks to replace the widely used phenylene or polycyclic aromatic groups for enhancing the stacking interactions. In this way, a series of metalla[2]catenanes, linear metalla[3]catenanes and BRs based on an organometallic half-sandwich unit were realized by high-yield and one-step processes (Fig. 1). The bithiophenyl groups effectively enhance the strength of the inter-ring interactions and play a crucial role in the formation of these homogeneous interlocked molecules.

Generally, macrocycle or cages based on coordination self-assembly commonly bear several positive charges and are more suitable for encapsulation of electroneutral or electron-rich guests than of electron-poor cations because of the Coulombic repulsion between host and guest. However, the authors took advantage of strong electrostatic interactions between electron-rich (π-donor, D) and electron-deficient (π-acceptor, A) aromatic groups to overcome the Coulombic repulsion and realized the introduction of the electron-deficient methylviologen cation into cationic metallarectangles. Following this logic, a heterogeneous D-A ring-in-ring complex was successfully prepared by threading a cationic metallarectangle based on an electron-deficient naphthalenediimide (NDI) unit inside a metallarectangle based on electron-rich bithiophenyl groups. On this basis, the method for a heterogeneous D-A ring-in-ring complex was extended by use of a metallarectangle based on a pyrenyl group.

**Figure 1.**
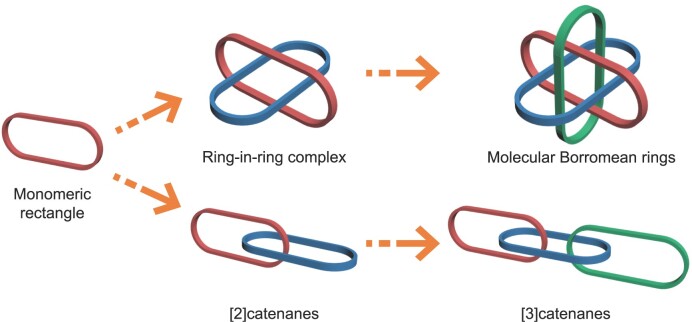
Various interlocked structures: monomeric macrocycle; ring-in-ring complex; Borromean rings; [2]catenanes; linear [3]catenanes.

The work of Prof. Guo-Xin Jin’s research group provided new ideas for designing the complicated topologic interlocked structures and will help the understanding of coordination self-assembly and boost the development of organometallic assemblies. It demonstrates how the formation of the resulting structures can be well controlled and modulated by the incorporation of various types of noncovalent bonding interactions into complex self-assembly systems. Moreover, this self-assembly process takes us one step past efficient construction to seeking possible applications through rational design.

***Conflict of interest statement*.** None declared.

## References

[1] Forgan RS , Sauvage JP, Stoddart JF. Chem Rev 2011; 111: 5434–64. 10.1021/cr200034u 21692460

[2] Gil-Ramirez G , Leigh DA, Stephens AJ. Angew Chem Int Edit 2015; 54: 6110–50. 10.1002/anie.201411619 PMC4515087 25951013

[3] Gao WX , Feng HJ, Guo BB et al. Chem Rev 2020; 120: 6288–325. 10.1021/acs.chemrev.0c00321 32558562

[4] Lu Y , Zhang HN, Jin GX. Acc Chem Res 2018; 51: 2148–58. 10.1021/acs.accounts.8b00220 29987929

[5] Lu Y , Liu D, Lin YJ et al. Natl Sci Rev 2020; 7: 1548–56. 10.1093/nsr/nwaa164 PMC8290965 34691487

